# Growth Hormone Secretagogue Receptor Dimers: A New Pharmacological Target^1^,^2^,^3^

**DOI:** 10.1523/ENEURO.0053-14.2015

**Published:** 2015-04-24

**Authors:** Martin Wellman, Alfonso Abizaid

**Affiliations:** Department of Neuroscience, Carleton University, Ottawa, Ontario, Canada, K1S 5B6

**Keywords:** G protein-coupled receptor, ghrelin, growth hormone secretagogue receptor, pharmacology, receptor dimerization

## Abstract

The growth hormone secretagogue receptor (GHSR1a), the target of the ghrelin peptide, is widely distributed throughout the brain, and, while studies have often reported very low or absent levels of central ghrelin, it is now known that GHSR1a, even in the absence of a natural ligand, has physiological roles. Not only do these roles originate from the receptor’s constitutive activity, but recent data indicate that GHSR1a dimerizes with a wide array of other receptors. These include the dopamine 1 receptor (D1R), the dopamine 2 receptor (D2R), the melanocortin-3 receptor (MC3R), the serotonin 2C receptor (5-HT_2C_), and possibly the cannabinoid type 1 receptor (CB1). Within these dimers, signaling of the protomers involved are modified through facilitation, inhibition, and even modification of signaling pathways resulting in physiological consequences not seen in the absence of these dimers. While in some cases the ghrelin peptide is not required for these modifications to occur, in others, the presence is necessary for these changes to take effect. These heterodimers demonstrate the broad array of roles and complexity of the ghrelin system. By better understanding how these dimers work, it is hoped that improved treatments for a variety of disorders, including Parkinson’s disease, schizophrenia, addiction, obesity, diabetes, and more, can be devised. In this review, we examine the current state of knowledge surrounding GHSR heterodimers, and how we can apply this knowledge to various pharmacological treatments.

## Abstract (precis)

The growth hormone secretagogue receptor, a key component of the ghrelin system, is important in many processes, including feeding behavior, stress, and reward. Recent data have revealed a variety of receptors that show the ability to dimerize with the ghrelin receptor, resulting in signal modulation, alterations in signaling cascades, and changes in trafficking and internalization of both protomers of the dimer complex.

## Significance Statement

The growth hormone secretagogue receptor, a key component of the ghrelin system, is important in many processes, including feeding behavior, stress, and reward. Recent data have revealed a variety of receptors that show the ability to dimerize with the ghrelin receptor, resulting in signal modulation, alterations in signaling cascades, and changes in trafficking and internalization of both protomers of the dimer complex. Here, we summarize the state of knowledge surrounding ghrelin receptor dimerization, along with potential treatments that arise from this knowledge for a variety of disorders, demonstrating the importance of understanding these dimer complexes.

## Introduction

It has been 15 years since the discovery of ghrelin, a peptide hormone related to, among others, energy balance, feeding, stress, anxiety, and reward. Since its discovery, this hormone has led to a staggering number of studies, particularly in response to the current obesity epidemic found throughout modern society. These studies have been prompted by ghrelin’s role as an orexigenic peptide, leading to increased feeding and adiposity, along with its role in diabetes. In general, three components of the ghrelin system have been targeted in the search for an elusive pharmacological compound that will contribute to weight loss. These are: (1) ghrelin itself, particularly its active esterified form: acylated ghrelin; (2) ghrelin O-acyltransferase (GOAT), the enzyme that acylates ghrelin, thus allowing it to bind to its receptor; and finally (3) the ghrelin receptor: GHSR1a.

While these three components are all directly related to ghrelin, one cannot deny the complex interrelationships between ghrelin and the multitude of other systems beyond simply the typical endocrine and feeding effects of this peptide. In line with this is an important concept in the study of G protein-coupled receptors (GPCRs): the idea of receptor oligomerization, in which GPCRs interact with other GPCRs in close proximity, resulting in physical association with possible conformational changes as well as changes in intracellular signaling and/or ligand binding. On top of this, dimerization may result in facilitation or inhibition of the protomers involved, as well as changes in surface expression, internalization, and trafficking.

Not only is ghrelin linked to obesity and diabetes, but studies have also shown a role in stress, anxiety, depression, and more (Lutter et al., 2008; Zheng et al., 2009; Patterson et al., 2010). Due to its roles in such a broad array of systems, targeting the ghrelin receptor with agonists or antagonists will not only affect the phenomenon of interest, such as feeding, but will also affect the other systems in which ghrelin is involved, leading to unwanted side effects. The ability to target only a subset of ghrelin receptors is a desirable goal that would hopefully minimize side effects and maximize treatment. By understanding the roles of receptor dimers, one would hope that designed drugs that target a specific dimer would be beneficial in pharmacological treatment. Such a dimer involving GHSR would be expected to have a more specific role than that of the ghrelin receptor in general. In addition to this, understanding how receptors are modified with dimerization can help us better understand how to treat certain disorders, providing hints for combinations of drugs that may synergistically amplify the desired effect or allow for a reduced dosage of certain medications with the goal of reducing intensity of side-effects.

In this review, we will examine the phenomenon of receptor dimerization with a focus on the role of the ghrelin receptor in such dimers and how this knowledge can aid in devising improved treatments for such disorders as Parkinson’s disease, schizophrenia, obesity, depression, and diabetes. At present, dimers between the GHSR1a and the D1R, D2R, 5-HT_2C_, MC3R, and possibly the CB1 receptors have been identified, and no doubt more interactions will be found further down the road.

## A Short History of GPCR Oligomerization

It has been known for over three decades that many membrane proteins exist as dimers or higher-order oligomeric structures (Klingenberg, 1981). Despite this, it was debated whether GPCRs in particular act solely as monomeric structures or if they oligomerize to form complexes with different characteristics from its constituent parts. While crosstalk between signaling cascades was acknowledged, actual allosteric interactions remained an area of debate. Some data suggests that the area accessible to G-proteins on the receptor is insufficient for transduction of the receptor signal to the relatively large trimeric G-protein complex (Park et al., 2004). Similarly, many non-GPCR membrane proteins also appear too small to interact with their proper ligands. Such proteins include the Na^+^-K^+^ ATPase, the ADP/ATP carrier, and the glucose carrier (Klingenberg, 1981). While dimerization and oligomerization of integral membrane proteins remained a generally accepted concept, translating this to GPCRs remained elusive (Klingenberg, 1981).

The first identified dimerization of GPCRs involved homodimers. These included the δ-opioid (Cvejic and Devi, 1997), metabotropic glutamate (Romano et al., 1996), and β_2_ adrenergic (Hebert et al., 1996) receptors. Shortly after the discovery of these homodimers, Jones et al. (1998) identified the first GPCR heterodimer and described it as an obligate heterodimer, consisting of the complex formed by a GABA_B_R1 and a GABA_B_R2 protomer (Jones et al., 1998; Kaupmann et al., 1998; White et al., 1998). These two receptors were believed to interact through conserved coiled-coil domains in the intracellular C-termini, a phenomena that was later verified (Kammerer et al., 1999; Kuner et al., 1999). Margeta-Mitrovic et al. (2000) identified a C-terminal RSRR retention motif in GABA_B_R1 that retains the receptor on the endoplasmic reticulum, thus preventing surface expression. Interaction with a GABA_B_R2 through each partners’ C-terminal coiled-coils masks the retention motif, thus allowing surface expression of the heterodimer. In addition, interaction with the intracellular G-protein for signal transduction appeared to be reliant on the GABA_B_R2 protomer, with ligand binding reliant on the GABA_B_R1 protomer (Margeta-Mitrovic et al., 2000; Robbins et al., 2001). These data demonstrate the obligate heterodimer nature of the GABA_B_ receptor.

## Detecting Oligomerization

There is some ambiguity when it comes to defining and identifying oligomerization. Often, receptors are coexpressed on the same neuron but do not form a multimeric complex with unique characteristics. While signaling may overlap and receptors may be in close proximity, the group of receptors might not necessarily generate a functional receptor complex. In order to remove ambiguity, the International Union of Basic and Clinical Pharmacology suggested a set of requirements in order for a group of receptors to be considered as part of an oligomeric complex. We refer the reader to Pin et al. (2007) for a detailed description of these requirements.

One of the most common techniques in detecting oligomerization is coimmunoprecipitation, where pulling down one protomer also pulls down its dimer partner. There are, however, drawbacks to this technique. For instance, coimmunoprecipitation cannot be used to examine interactions in living cells in real time. In addition, aggregation as a result of the solubilization step is a common problem, resulting in false positives (Szidonya et al., 2008). Another source of false positives is the high levels of receptor expression typically used with this technique, which can lead to nonphysiological dimerization simply as a result of overexpression and crowding (Szidonya et al., 2008). In contrast, dimers may be disrupted during preparation of the samples and give false negatives (Szidonya et al., 2008). Finally, raising antibodies directly against GPCRs is a task that has proven to be quite difficult (Fredholm et al., 2007), although tagging alternatives are available.

**Figure 1:**
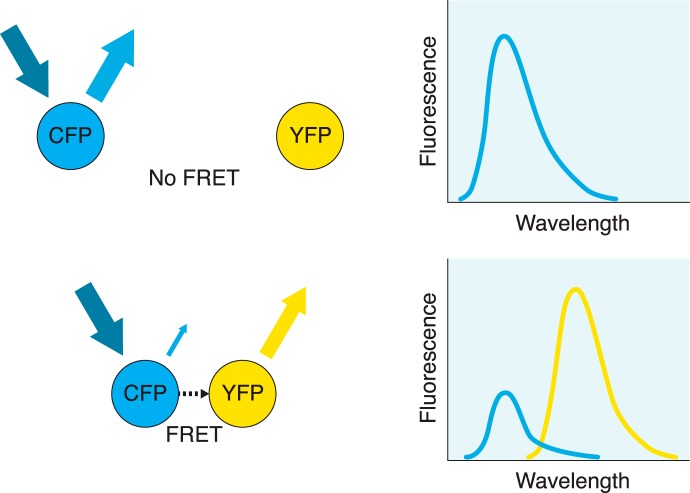
Fluorescence resonance energy transfer. In this FRET example, cyan fluorescent protein (CFP) acts as the donor and yellow fluorescent protein (YFP) as the acceptor. When the two fluorophores are separated by a considerable distance, exposing the sample to light with the excitation frequency for CFP results in an emission spectrum corresponding to CFP only, with no contribution from the acceptor (top). When the two fluorophores are nearby (typically in the range of 10 to 100 Å), exposing the sample to the same light results in a nonradiative energy transfer from CFP to the nearby YFP acceptor, causing YFP to emit at its emission frequency (bottom). At the same time, due to the transfer of energy, emission from CFP is considerably reduced. Detection of YFP emission indicates that fluorescence resonance energy transfer has occurred between the two fluorophores as a result of their close proximity. By fusing these fluorophores to the receptors of interest, dimerization can be implied if fluorescence resonance energy transfer is observed.

A technique that avoids many of the problems of coimmunoprecipitation is resonance energy transfer (RET) and its derivative techniques (Fig. 1). Perhaps one of its most powerful advantages is its ability to measure dimerization in real-time in living cells. While there are many techniques related to RET, they all involve the nonradiative transfer of energy (on the nanoscale range) between a donor and a nearby acceptor, which have been fused to the two receptors of interest. This transfer is measured by examining the emission of energy from the acceptor after it has accepted the transfer from the donor. For example, in the case of fluorescence resonance energy transfer (FRET), the donor is excited by light of a certain wavelength. If the donor is in close proximity to the acceptor, this energy is transferred and then emitted at a characteristic wavelength by the acceptor, which can be measured by scanning spectroscopy, a microplate reader, or microscopy. In the case of bioluminescence resonance energy transfer (BRET), a donor such as the enzyme Renilla luciferase is used.

While RET-based techniques are considerably more powerful than coimmunoprecipitation, there are some disadvantages to this technique. Of particular importance is the requirement for a proper orientation of the dipoles in order for the energy transfer to occur (Szidonya et al., 2008). Along with this, the efficiency of the transfer is inversely proportional to the sixth power of the distance between the dipoles, demonstrating extreme sensitivity of the technique to distance (Esen-Danaci et al., 2008). These restrictions require that the donor and acceptor have considerable flexibility in movement in order to acquire the correct orientation and distance.

Along with FRET and BRET, there are other derivatives of RET. For a more detailed discussion of coimmunoprecipitation and RET as they relate to GPCR oligomerization, we refer the reader to Szidonya et al. (2008).

### Signaling and distribution of the GHSR1a

The GHSR1a was first identified in 1996 by a team of researchers who were looking for a way to improve growth hormone release (Howard et al., 1996), and this discovery was followed by the identification of its natural ligand, ghrelin, a peptide hormone produced by the stomach (Kojima et al., 1999). Interestingly, ghrelin requires acylation on its serine-3 residue by the enzyme GOAT in order for the peptide to bind to GHSR1a (Kojima et al., 1999; Gutierrez et al., 2008; Yang et al., 2008).

Signal transduction of GHSR1a primarily occurs through the G_q/11_ pathway (Camiña et al., 2007). This pathway involves activation of phosphatidylinositol phospholipase C (PI-PLC), phosphatidylinositol 4,5-bisphosphate (PIP_2_) and inositol trisphosphate (IP_3_), ultimately resulting in mobilization of intracellular Ca^2+^ stores (Camiña et al., 2007). In addition to the G_q/11_ pathway, the G_i/o_ pathway has also been shown to be recruited by the ghrelin receptor. In rat and human tissue, high signals for GHSR1a mRNA are found expressed in several hypothalamic nuclei, the pituitary, and the dentate gyrus of the hippocampus, with additional yet reduced signals in CA2 and CA3 of the hippocampus, the substantia nigra, the ventral tegmental area, and the median raphe nuclei (Guan et al., 1997; Willesen et al., 1999; Gnanapavan et al., 2002; Zigman et al., 2006). Of particular interest is the arcuate nucleus, an area more open to peripheral signals due to the reduced blood−brain barrier at the median eminence. Indeed, many labs have measured high levels of GHSR1a mRNA in the arcuate nucleus (Howard et al., 1996; Guan et al., 1997; Tannenbaum et al., 1998; Willesen et al., 1999; Mitchell et al., 2001; Zigman et al., 2006). This area’s important role in feeding is undeniable, with extensive connections to all other areas of the hypothalamus. While GHSR1a activity in the hypothalamus is likely heavily involved in homeostatic mechanisms, its presence in extra hypothalamic regions, including a number of limbic and midbrain structures like the ventral tegmental area (VTA), suggest that this receptor is associated in processes associated with learning and motivation (Abizaid et al., 2006; Abizaid and Horvath, 2008). Nevertheless, the entry of peripheral ghrelin into the brain is limited and potentially binds only to areas of the brain that are less protected by the blood−brain barrier like the arcuate and the area postrema in the brain stem (Banks et al., 2002; Cabral et al., 2013). Furthermore, evidence for a central source of ghrelin remain questionable, with only very low levels being found in the hypothalamus (Hosoda et al., 2000; Cowley et al., 2003). Without the natural ligand for the GHSR1a in many parts of the brain, the various roles of this receptor centrally remain uncertain.

Studies identifying a high constitutive activity of the ghrelin receptor (Holst et al., 2003; Damian et al., 2011) suggested that expression of GHSR1a itself, without the need for binding, could have physiological significance. Regulation of this constitutive activity, either through allosteric interactions with other proteins, regulation of receptor internalization and trafficking, or modulation of transcription and translation, became an important area of research. In the absence of ghrelin, GHSR1a shows 50% of its maximal ligand-stimulated activity through the G_q/11_ pathway (Holst et al., 2003). It is likely that overall changes in constitutive activity, in the absence of ghrelin, provide sufficient modulation of signaling pathways to demonstrate an important physiological effect. In line with this idea is a genetic study identifying a missense mutation, A240E located in the second extracellular loop, resulting in reduced constitutive activity and leading to familial short stature (Pantel et al., 2006). Importantly, this mutation did not display reduced ghrelin binding. While the phenotype of this mutation may be due to both peripheral and central actions, it demonstrates the importance of GHSR1a’s constitutive activity. Furthermore, GHSR1a’s constitutive activity has opened the door for investigation into the effects of inverse agonists, in particular [D-Arg^1^, D-Phe^5^, D-Trp^7,9^, Leu^11^]-substance P (SP-analog), which has in turn provided hints to the structure and epitopes of the ghrelin receptor.

Despite these findings, the high levels of GHSR1a expression used in experiments identifying GHSR1a’s constitutive activity may be the source of this unusually high basal activity. When expressed at the low levels representative of *in vivo* amounts, at least one laboratory has found that basal activity was not detectable (Kern et al., 2012). Furthermore, at the high expression levels used, GHSR1a homodimerization may be artificially enhanced. Whether homodimerization is required for constitutive activity is unknown, although GHSR1a homodimers have been detected. Indeed, while examining dimerization between the D2 receptor and GHSR1a, Kern et al. (2012) observed a FRET signal when CLIP-GHSR1a and SNAP-GHSR1a are overexpressed. Additionally, Jiang et al. (2006) observed GHSR1a homodimers in a HEK293-derived cell line by examining the BRET signal for GHSR1a-GFP/GHSR1a-Rluc, with the ratio displaying a hyperbolic shape, suggesting dimerization rather than crowding. As Schellekens et al. (2013) put it, the GHSR1a is quite a “promiscuous” receptor, not only with other receptors but also with itself.

Data from Rediger et al. (2011), however, suggest that the constitutive activity of GHSR1a is not the result of dimerization. Using two naturally occurring mutations of GHSR1a that demonstrate reduced constitutive activity, Rediger et al. (2011) were still able to detect homodimerization. Furthermore, data from Holst et al. (2004) suggest that constitutive activity depends on several residues on the inner face of GHSR1a, which seem unlikely to be locations important for the dimerization interface. While this constitutive activity alone gives support to a role of GHSR1a centrally where only extremely low levels of ghrelin have been found (Hosoda et al., 2000; Cowley et al., 2003), more recent findings of GHSR1a heterodimerization reveal a whole slew of roles that the receptor plays in the brain, some of which do not require the presence of the ghrelin peptide. Understanding these dimers will provide insight into possible pharmacological interventions.

### GHSR1a dimerization with D1- and D2-like dopamine receptors

The interaction between GHSR and dopamine receptors was first hypothesized given the coexpression of these receptors at a number of sites, including a number of brain regions associated with food intake and reward-seeking behaviors (Guan et al., 1997). The dopamine system is one of the most studied neurotransmitter systems, with dopamine being one of the earliest identified neurotransmitters due in large part to its pivotal role in the reward system, a system often described as being essential to all forms of life. In feeding behavior, enhanced dopaminergic activity induces rewarding effects and also enhances memory formation for events associated with reward (White, 1988, 1989; Centonze et al., 2001; Wise, 2006; Wise, 2008). One would intuitively suspect that ghrelin and dopamine interact with each other.

The group of dopamine receptors has two families: D1-like, which includes D1R and D5R, and D2-like, which includes D2R, D3R, and D4R. Jiang et al. (2006) demonstrated that, in the presence of both dopamine and ghrelin, the ghrelin receptor amplifies dopamine-induced cAMP accumulation via D1R. Subsequently, Kern et al. (2012) demonstrated that in the absence of ghrelin, GHSR1a oligomerizes with D2R and that this oligomerization is required for D2R’s anorexigenic effects.

### GHSR1a dimerization with the D1R amplifies D1R signaling

The D1 receptor has been localized to various regions of the brain, with an array of different functions, including but not limited to the substantia nigra and caudate-putamen (motor activity) (Double and Crocker, 1995; Centonze et al., 2003), prefrontal cortex and hippocampus (cognition, learning, and memory) (Sawaguchi et al., 1990; Sawaguchi and Goldman-Rakic, 1994; Williams and Goldman-Rakic, 1995; Otmakhova and Lisman, 1996; Matthies et al., 1997; Seamans et al., 1998; Granado et al., 2008), nucleus accumbens and olfactory tubercle (reward) (Kurumiya and Nakajima, 1988; Nakajima, 1989; Ikemoto et al., 1997; Ikemoto, 2003), and hypothalamus (feeding and reward) (Nakajima and McKenzie, 1986; Chen et al., 2014).

**Figure 2:**
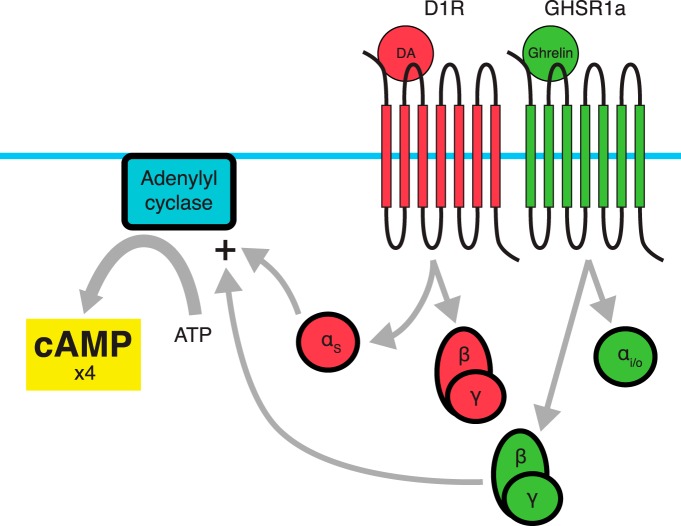
Dimerization between D1R and GHSR1a. When dimerized with D1R, GHSR1a switches G-protein coupling from G_q/11_ to G_i/o_. Coadministration of a D1R agonist with a GHSR1a agonist leads to a fourfold amplification of D1R-associated cAMP accumulation. It is believed that the G_βγ_ subunit associated with GHSR1a adopts a stimulatory role on adenylyl cyclase activity due to the proximity of the α_S_ subunit derived from D1R’s trimeric G-protein.

Coexpression of GHSR1a and D1R has been reported in the cortex, hippocampal structures, substantia nigra, midbrain, and ventral tegmental areas (Jiang et al., 2006). In HEK293 cells expressing D1R and GHSR1a, both a BRET signal and coimmunoprecipitation indicate dimerization between the two receptors (Jiang et al., 2006). As summarized in Figure 2, coadministration of dopamine and ghrelin to these cells show a fourfold amplification of D1R-associated cAMP signaling, with this amplification requiring both receptors and both ligands (Jiang et al., 2006). These data demonstrate dimerization in areas associated with mood, learning, and memory. Interestingly, when ghrelin is administered alone to cells coexpressing D1R and GHSR1a, no increase in cAMP accumulation is observed, while typical levels of Ca^2+^ accumulation associated with GHSR1a activation occur (Jiang et al., 2006). Despite this, the PKC inhibitor bisindolylmaleimide I (Bis) does not affect cAMP augmentation within the D1/GHSR1a dimer, while pertussis toxin, an inhibitor of the G_i/o_ pathway, eliminates cAMP augmentation (Jiang et al., 2006). In addition to this, pertussis toxin administration in the absence of ghrelin does not affect dopamine-associated cAMP accumulation, which typically signals through Gα_s_ and Gα_olf_ (Missale et al., 1998; Neve et al., 2004). These data suggest that the synergy between GHSR1a and D1R is due to GHSR1a switching from Gα_q/11_ coupling to Gα_i/o_ coupling, a G-protein that is not associated with D1R or GHSR1a when expressed alone. In addition to this synergistic effect, cointernalization of D1R-GHSR1a is induced after stimulation by the D_1_ agonist 6,7-ADTN hydrobromide or GHSR1a agonists (Schellekens et al., 2013). In effect, GHSR1a as well as D1R agonists can terminate the effect of the dimer partner’s natural ligands through cointernalization.

An effective way to test the role of the D1R-GHSR1a dimer in behaviors associated with ghrelin would be by way of mutations in GHSR1a or D1R that may negatively affect heterodimerization. For example, in the GABA_B_ receptor, heterodimers associate through C-terminal parallel coiled-coil α-helices, and mutations in their primary structure can eliminate dimerization (White et al., 1998; Kammerer et al., 1999). A bioinformatics approach may help reveal more data regarding amino acid sequences involved in dimerization associated with GHSR1a, which may eventually lead to identification of polymorphisms that affect dimerization.

Jiang et al. (2006) proposed a molecular mechanism for the synergistic effect of D1R-GHSR1a dimerization on cAMP accumulation. In the case of D1R, G-protein activation involves dissociation of the Gα_s_ and Gβγ subunits. Gα_s_ stimulates membrane-associated adenylyl cyclase (AC) activity. The Gβγ subunit is believed to play a modulatory role, with a stimulating effect for AC2, AC4, and AC7, and an inhibiting effect for AC1 and AC8 (Jiang et al., 2006). Interestingly, the Gβγ subunit can only play a stimulatory role when associated with Gα_S_. In the case of the D1R-GHSR1a heterodimer, it is believed that GHSR1a switches G-proteins from Gα_q/11_ to Gα_i_. Dissociation of the Gβγ subunit from Gα_i_, which would normally inhibit cAMP accumulation through adenylyl cyclase, switches to a stimulatory role due to the activation induced by the nearby Gα_S_ activity.

### GHSR1a dimerization with the D2 receptor: molecular and behavioral effects

Like the D1 receptor, the D2 receptor has a broad distribution, with the two showing considerable overlap. This includes high levels of expression in the neostriatum, olfactory tubercle, substantia nigra, ventral tegmental area, and the nucleus accumbens (Meador-Woodruff et al., 1989). While the D1 receptor stimulates adenylyl cyclase activity, the D2 receptor inhibits it through a Gα_i_ pathway (Missale et al., 1998; Neve et al., 2004).

Dimerization between D2R and GHSR1a has been demonstrated using FRET, with strong signals originating in hypothalamic cultures (Kern et al., 2012). This dimerization appears to induce a switch in intracellular signaling cascades in which administration of a dopamine agonist (quinpirole) alone leads to a rapid increase in Ca^2+^ levels, an effect not observed in preparations expressing D2R in the absence of GHSR1a (Kern et al., 2012). This effect can be attenuated by administering the D2R-specific antagonist raclopride or the GHSR1a inverse agonist SP-analog (Kern et al., 2012).

**Figure 3:**
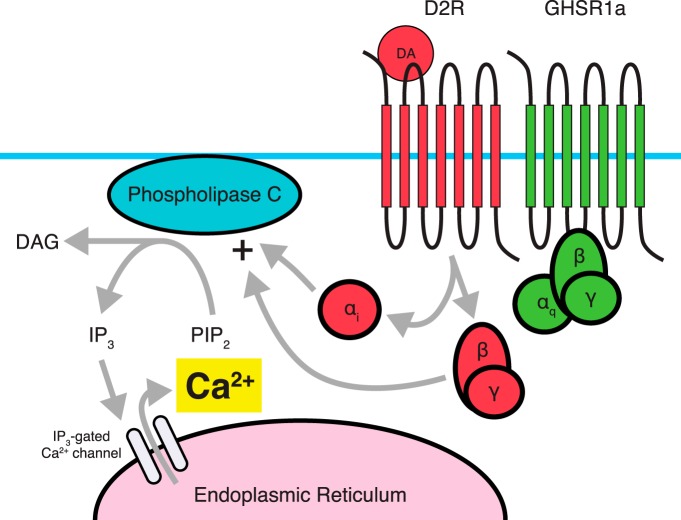
Dimerization between D2R and GHSR1a. Proposed signaling through D2R involves coupling to a G_i_ pathway, which typically does not involve intracellular Ca^2+^ accumulation from the endoplasmic reticulum. Dimerization with GHSR1a, in the absence of a ghrelin ligand leads to a PLC-dependent accumulation of Ca^2+^. D2R’s G_βγ_ subunit acts to stimulate PLC activity, and α_i_ coupling by D2R is also required for Ca^2+^ accumulation. In contrast, Gα_q_ activity associated with GHSR1a is not required for D2R-induced Ca^2+^ accumulation. It is believed that the D2R-GHSR1a dimer is responsible for the anorectic effects of D2R agonists such as cabergoline.

Using a variety of inhibitors of second messenger signaling molecules, Kern and colleagues (2012) identified the pathway responsible for D2R-induced Ca^2+^ mobilization when dimerized with GHSR1a. This pathway included PLC-dependent activation through Gα_i_ coupling, ultimately leading to release of Ca^2+^ from the endoplasmic reticulum via IP_3_ receptors (Fig. 3). Interestingly, by similarly inhibiting specific pathway components, it was shown that this signaling was dependent on Gβγ subunits derived from D2R’s Gα_i/o_, stimulating PLC activity. Furthermore, dimerization of D2R with a GHSR1a mutant lacking constitutive activity still displayed dopamine-induced Ca^2+^ mobilization, albeit considerably reduced, while two constitutively active mutants were absent in Ca^2+^ mobilization, suggesting that Ca^2+^ mobilization is independent of GHSR1a constitutive activity. In addition, inhibiting GHSR1a’s Gα_q_ using siRNA did not result in loss of dopamine-induced Ca^2+^ mobilization, but did significantly reduce ghrelin-induced Ca^2+^ mobilization. Overall, evidence suggests that GHSR1a’s constitutive activity is not required for the alteration in D2R-mediated signaling. Perhaps the most striking results obtained by Kern et al. are the behavioral data examining the interaction between D2R and the GHSR1a in mice (Vucetic and Reyes, 2010; Kern et al., 2012). Cabergoline, a D2R-selective agonist, produces a dose-dependent suppression of food intake in wild-type mice and in ghrelin KO mice, but has no effect on food intake in *GHSR KO* mice (Kern et al., 2012). These data clearly show that the anorexigenic effects of cabergoline depend on GHSR1a and not on ghrelin, providing more evidence that GHSR1a has a central role even in the absence of the ghrelin peptide.

While these data are fascinating, a natural question that arises is what happens if ghrelin levels are allowed to increase? This examination may be subject to debate in the face of extremely low central ghrelin levels. There is a possibility, however, that certain manipulations such as stress or food restriction/deprivation may cause an increase in central ghrelin levels. If one were to examine the effects of elevated ghrelin levels, some interesting questions may be posed. As discussed, it appears that the D2R-induced anorexigenic effect of dopamine depends on the presence of GHSR1a. Kern et al. (2012) found cross-desensitization within the D2R-GHSR1a heteromer. Specifically, pretreatment with the GHSR1a agonist MK-0677 or ghrelin for 30 min resulted in an attenuated response to dopamine, with 60-75% reduction in calcium mobilization. One way that this reduction might occur is through desensitization, disassociation, or cointernalization of the dimer. If ghrelin were to be present in the brain for a prolonged period of time, it would eventually attenuate the anorexigenic effect of D2 receptor through cross-desensitization. Interestingly, administration of the GHSR1a antagonist JMV2959 or the inverse agonist SP-analog also attenuate the anorexigenic effect caused by D2 agonists, but in this case, the attenuation appeared immediately. The more immediate effects of GHSR receptor antagonist administration on dopamine’s anorectic signaling deserves examination. During the period over which desensitization is being established, other signaling molecules and pathways may contribute to behavioral changes and examining the time frame of changes in the levels of relevant neurotransmitters may help tease apart the complexities associated with feeding regulatory systems.

### GHSR1a dimerization with the melanocortin-3 receptor amplifies MC3R and inhibits GHSR1a signaling

**Figure 4:**
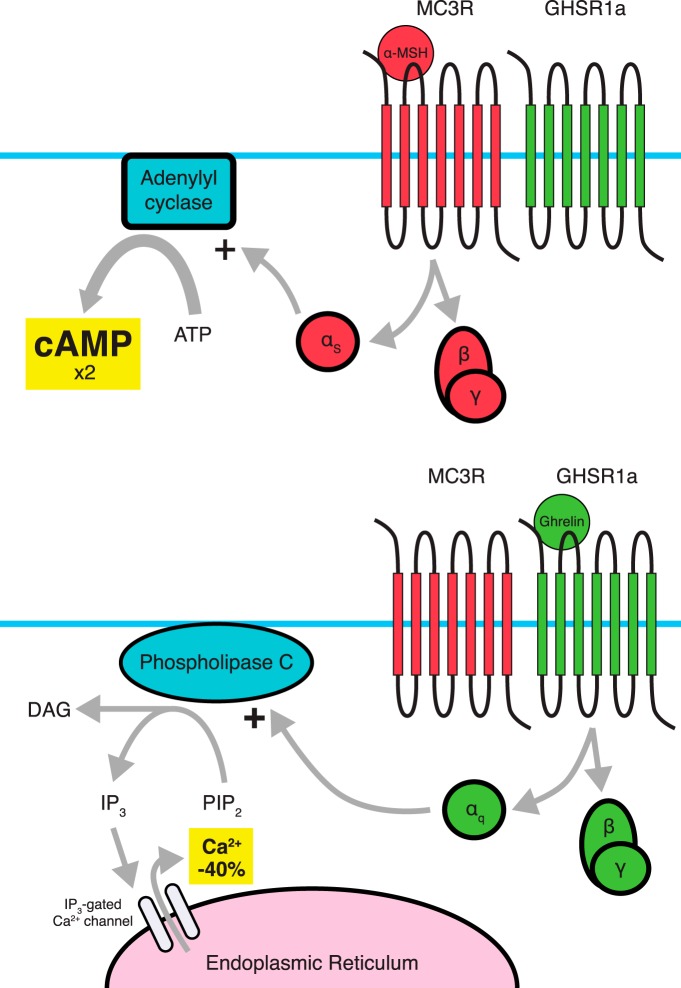
Dimerization between MC3R and GHSR1a results in amplification of MC3R signaling and attenuation of GHSR1a signaling. While it is believed that the pathways involved are not changed, changes in the amplitude of the signals occur. Dimerization with stimulation of MC3R leads to a twofold amplification of MC3R-induced cAMP accumulation (top), while the GHSR1a protomer shows a ligand-dependent as well as ligand-independent 40% reduction in Ca^2+^ accumulation when dimerized with MC3R (bottom). Amplification of MC3R signaling appears to be dependent on GHSR1a’s constitutive activity.

Within the arcuate nucleus, most GHSR1a-expressing neurons coexpress MC3R, while a much smaller proportion of MC3R-expressing neurons coexpress GHSR1a (Rediger et al., 2009). Within this overlapping expression, Rediger et al. (2009) have identified MC3R-GHSR1a dimers using FRET. Typical signaling through MC3R involves a Gα_s_ pathway leading to cAMP accumulation. Within this dimer, MC3R-associated cAMP accumulation is increased twofold while GHSR1a constitutive activity and ghrelin-induced activity, as measured by intracellular Ca^2+^ levels, are reduced by 40% (Fig. 4) (Rediger et al., 2009). Coadministration of α-melanocyte-stimulating hormone (α-MSH) with ghrelin does not affect these changes in signaling (Rediger et al., 2009).

Much of our knowledge surrounding the MC3R derives from studies involving *MC3R KO* animals, which demonstrate increased adiposity, reduced lean mass, and increased feed efficiency, with mildly reduced or absent changes in caloric intake and normal metabolic rates (Butler et al., 2000; Chen et al., 2000). In wild-type animals, MC3R activation has been associated with anorectic effects (Fan et al., 1997; Thiele et al., 1998; Cowley et al., 1999).

In the absence of MC3R, the inhibition on GHSR1a is absent, resulting in an increase in GHSR1a signaling. While this may explain in part the increase in fat mass in *MC3R^−/−^* animals, it does not explain the hypophagia sometimes found in these animals (Chen et al., 2000). If one ignores the traditional caveats of developmental adaptions in knock-out animals, this suggests that ghrelin’s ability to increase feeding, particularly of food with high fat content, might be dependent on MC3R, with the possibility that ghrelin-induced, feeding-independent adiposity does not. Indeed, it has been shown that *MC3R KO* animals do not display ghrelin-induced hyperphagia (Shaw et al., 2005). Complicating matters, signaling downstream of GHSR1a involves amplification of agouti-related peptide (AgRP), which is an inverse agonist of MC3R (Breit et al., 2006; Tao et al., 2010). To the best of our knowledge, no study has examined how AgRP affects the MC3R-GHSR1a heterodimer.

Additionally, when HEK293 cells are cotransfected with MC3R and a GHSR1a mutant showing impaired or absent constitutive activity, heterodimerization still occurs but amplification of α-MSH-induced cAMP accumulation is lost, suggesting that the twofold increase in cAMP accumulation depends on GHSR1a basal activity (Rediger et al., 2011). This effect of GHSR1a’s constitutive activity again demonstrates that the receptor’s basal activity may show physiological consequences without the need for the ghrelin peptide.

In examining the GHSR1a-MC3R dimer, Schellekens et al. (2013) demonstrated that activation by ghrelin or the synthetic ghrelin agonist MK-0677 causes an increase in internalized dimers, yet no significant changes are observed when treated with the MC3R agonist [Nle^4^,D-Phe^7^]-α-MSH. Interestingly, under basal conditions when expressed with MC3R, a higher level of GHSR1a internalization is found (Schellekens et al., 2013). These additionally internalized receptors are for the most part dimerized with MC3R (Schellekens et al., 2013), suggesting that dimerization with MC3R may not only lead to reduced efficiency of GHSR1a signaling, but also to reduced surface expression of the receptor.

While GHSR1a constitutive activity is required for amplification of MC3R signaling, it has minimal effect on GHSR1a-MC3R dimer internalization, as treatment with the GHSR1a inverse agonist SP-analog only results in a small, nonsignificant reduction in internalization (Schellekens et al., 2013). However, this mild reduction is enough to demonstrate a significant increase in cointernalization in response to [Nle^4^,D-Phe^7^]-α-MSH when compared to the SP-analog treatment (Schellekens et al., 2013). In contrast, treatment with [Nle^4^,D-Phe^7^]-α-MSH does not result in a significant increase in cointernalization when compared to controls (Schellekens et al., 2013).

Nevertheless, both Schellekens et al.’s (2013) and Rediger et al.’s (2009) studies demonstrate a significant loss of both ligand-independent and ligand-dependent GHSR1a-mediated Ca^2+^ accumulation when the GHSR1a is dimerized with the MC3R. These data suggest that GHSR1a signaling may be compromised in areas such as the arcuate nucleus, where high levels of both MC3R and GHSR1a expression are reported (Rediger et al., 2009). The conditions that lead to this dimerization, as well as the specific cell groups showing coexpression of both GHSR1a and MC3R, remain to be fully characterized and should be examined in detail. In particular, looking at the melanocortin system by way of examining which neurons show dimerization between MC3R and GHSR1a may add a new level of complexity in the mechanisms underlying the melanocortin feeding system.

### GHSR1a dimerization with 5-HT_2C_ results in reduced GHSR1a signaling

**Figure 5:**
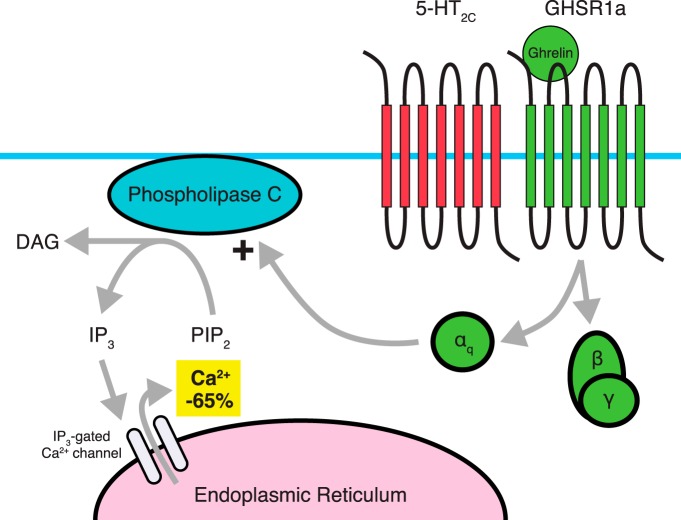
Dimerization between 5-HT_2C_ and GHSR1a. When dimerized with 5-HT_2C_. GHSR1a displays a 65% reduction in ghrelin-induced Ca^2+^ accumulation, with this effect not requiring the presence of a 5-HT_2C_ ligand. While changes in serotonergic signaling associated with the dimer through 5-HT_2C_ have not yet been observed, cross-desensitization, cross-sensitization, and cointernalization do occur.

Recently, the 5-HT_2C_ receptor has also been identified as a dimerization partner of GHSR1a (Fig. 5). Like the GHSR1a, the 5-HT_2C_ serotonin receptor signals through a Gα_q_ pathway, leading to Ca^2+^ accumulation (Schellekens et al., 2013). Paradoxically, stimulation of the 5-HT_2C_ receptor results in decreased food intake and adiposity, while mutations to the gene encoding for this receptor result in obesity (Sargent et al., 1997; Martin et al., 1998; Nonogaki et al., 1998; Somerville et al., 2007; Garfield and Heisler, 2009). One proposed mechanism of action is that stimulation of 5-HT_2C_ receptors in pro-opiomelanocortin (POMC) neurons within the arcuate nucleus of the hypothalamus leads to increased release of α-MSH (Garfield and Heisler, 2009).

Recently, Schellekens et al. (2013) examined the possibility of a 5-HT_2C_-GHSR1a heterodimer that explained the interaction between serotonin and ghrelin reported in the literature. This included, for instance, negative feedback from 5-HT_2C_ receptor agonism onto ghrelin levels in overnight fasted mice (Nonogaki et al., 2006); the inhibition of serotonin release by ghrelin in rat hypothalamic synaptosomes (Brunetti et al., 2002); the effects of serotonin on reward-related behaviors (Higgins and Fletcher, 2003; Alex and Pehek, 2007); the ability of serotonin to block ghrelin’s orexigenic effects (Currie et al., 2010); and some of the overlapping expression profiles of 5-HT_2C_ and GHSR1a (Abramowski et al., 1995; Guan et al., 1997; Cowley et al., 2003; Zigman et al., 2006). This group demonstrated that dimerization occurs between 5-HT_2C_ and GHSR1a. In particular, pretreatment with the GHSR1a inverse agonist SP-analog results in cross-sensitization to the 5-HT_2C_ response in cells coexpressing GHSR1a and 5-HT_2C_ (Schellekens et al., 2013). Typically, in cells expressing GHSR1a alone, pretreatment with SP-analog results in increased surface expression of GHSR1a and increased sensitivity. Additionally, cells expressing both receptors show a 65% attenuation of Ca^2+^ accumulation in response to ghrelin or the ghrelin agonist MK-0677, with this attenuation being mostly restored by the 5-HT_2C_ specific antagonist RS102221. Interestingly, beyond sensitivity and internalization effects, no immediate modification of 5-HT_2C_ signaling occurs in any of these situations (Schellekens et al., 2013).

When exposed to ghrelin, an increase in 5-HT_2C_-GHSR1a dimer cointernalization occurs (Schellekens et al., 2013). While exposure to the synthetic GHSR1a agonist MK-0677 results in an increase in internalization, this increase does not reach significance when compared to control, whereas it does reach significance compared to SP-analog (Schellekens et al., 2013). Schellekens et al. (2013) suggest that the lack of significance is likely due to the high levels of cointernalization under control conditions. Overall, it appears that GHSR1a dimerization with 5-HT_2C_ reduces overall ghrelin signaling, and hence may reduce feeding behavior.

## Possible Dimerization with the Cannabinoid Receptor Type 1

While dimerization between the endocannabinoid receptor CB1 and GHSR1a has not been examined directly, there is evidence suggesting that such dimerization may occur. It has been previously shown that both ghrelin (via GHSR1a) and the cannabinoids (via CB1) have orexigenic effects (Williams et al., 1998; Inui, 2001), with both increasing AMP-activated protein kinase (AMPK) activity in the hypothalamus and reducing it in the liver and adipose tissue (Kola et al., 2005). Interestingly, Kola and colleagues (2008) demonstrated that ghrelin’s orexigenic and stimulating AMPK effects in the hypothalamus are lost in *CB1 KO* or mice treated with the CB1 antagonist Rimonabant. Patch-clamp electrophysiology of parvocellular neurons of the paraventricular nucleus also indicated that application of the CB1 antagonist AM251 eliminated ghrelin’s electrophysiological effects (Kola et al., 2008). In addition to this, ghrelin increases endocannabinoids in the hypothalamus of wild-type but not *CB1 KO* mice (Kola et al., 2008). Conversely, administration of HU210 (a CB1 agonist) significantly stimulated hypothalamic AMPK activity and inhibited visceral fat and liver AMPK activity in wild-type mice, but these effects were lost in *GHSR KO* animals (Lim et al., 2013). Additionally, while no significance was found, a trend to an increase in food intake with intraperitoneal injection of HU210 was observed; this trend was not visible in *GHSR KO* mice (Lim et al., 2013).

These data provide preliminary evidence of the possible dimerization between CB1 and GHSR1a, as suggested by Lim et al. (2013). Despite this, one must also consider the possibility that this dependence is not due to dimerization. In one model suggested by Kola and Korbonits (2009), endocannabinoid synthesis is placed downstream of GHSR1a activation, with *GHSR KO* animals having no endocannabinoid-associated response to ghrelin administration. However, the bidirectional dependency between CB1 and GHSR1a presented in the study is suggestive of a receptor interaction. Nevertheless, it is possible that positive feedback between the two systems is required for ample Ca^2+^ accumulation to occur to induce increases in AMPK and affect feeding. In addition to ghrelin losing its feeding effect in *CB1^−/−^* animals, neuropeptide Y (NPY) has also been shown to lose its feeding effect in *CB1^−/−^* animals (Poncelet et al., 2003). In Kola’s model, NPY signaling occurs downstream of both GHSR1a and CB1. Whether a possible positive feedback loop includes NPY as well is not known, but the system is no doubt more complicated than presented here.

### General treatment approaches

**Figure 6:**
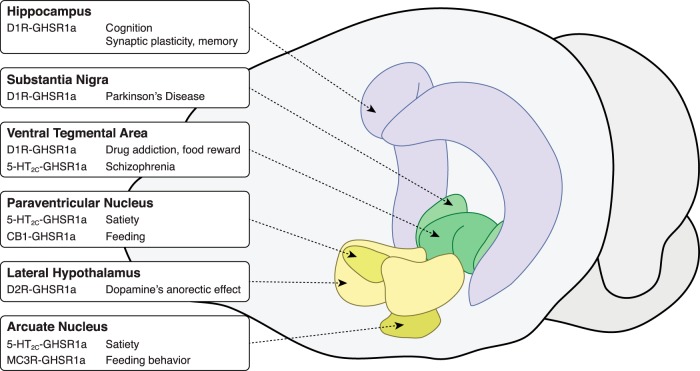
Selected areas of interest involving possible GHSR1a dimers along with postulated roles/treatments. Brain figure adapted from the Allen Mouse Brain Atlas (Lein et al., 2007).

As summarized in Figure 6, the dimer partners discussed in this review provide hints as to possible treatment targets for various disorders and conditions. In some cases, these treatments could involve the use of multiple drugs to provide a synergistic effect, amplifying the effect seen when only one drug is administered. In others, supplementing a treatment with an amplifying agent may have beneficial off-target effects beyond synergy. In such cases, not only do these treatments amplify neurotransmitter receptor signaling, but they may have other possible effects such as reduction in depression, neuroprotection, and stimulation of appetite—all effects that may relate to the effects on GHSR alone and not the dimer. The identification of GHSR heterodimers certainly opens the door for potential new treatments and treatment adjuvants that could improve a number of chronic psychiatric and metabolic conditions.

### Targeting the D1R-GHSR1a dimer for treatment of Parkinson’s disease

A standard treatment for Parkinson’s disease typically involves the use of drugs that restore dopamine concentrations from the low levels caused by the loss of dopaminergic neurons in the substantia nigra (Mercuri and Bernardi, 2005). The coexpression of the ghrelin and D1 receptors in this area suggests that supplementing this treatment, or perhaps even replacing it, with a ghrelin mimetic may help amplify D1 signaling of the remaining dopaminergic activity. The addition of a ghrelin mimetic may also aid through other ghrelin-related actions, including neuroprotection. Indeed, several studies have examined the beneficial effects of ghrelin in treatment for Parkinson’s disease. In particular, ghrelin and its receptor have been shown to protect dopaminergic function from 1-methyl-4-phenyl-1,2,3,6-tetrahydropyridine (MPTP)-induced degeneration in the substantia nigra pars compacta and the dorsal striatum (Andrews et al., 2009; Moon et al., 2009). Andrews and colleagues (2009) demonstrated that MPTP-induced dopaminergic dysfunction is amplified in *GHSR KO* animals, and selective re-expression of GHSR1a in catecholaminergic neurons of *GHSR KO* animals reduced this degeneration. These data suggest that ghrelin may aid in two ways: (1) by amplifying the remaining dopaminergic signaling through the D1R-GHSR1a dimer, and (2) by providing neuroprotective effects that may aid in slowing the progression of further dopaminergic degeneration, which may be the result of GHSR signaling alone.

### Treating drug addiction and preventing relapse with ghrelin antagonists

There is now substantial evidence suggesting that GHSR located in the mesolimbic dopaminergic system is associated with the amplification of reward-seeking behaviors, including those associated with drugs of abuse (Abizaid, 2009; Dickson et al., 2011; Suchankova et al., 2013; Wellman et al., 2013; Leggio et al., 2014). Given the described D1R-GHSR1a dimer, one could propose that GHSR antagonism reduces the reinforcing effects of drugs of abuse like alcohol, nicotine, and cocaine by reducing D1 receptor signaling produced by the D1R-GHSR1a dimer. Enhanced D1 receptor signaling would also explain why addicts are more prone to relapse when stressed or hungry, both conditions that augment ghrelin secretion and sensitivity (Carroll, 1985; Brown et al., 1995; Shalev et al., 2000; Shalev et al., 2001; Goeders, 2003; Sinha et al., 2006). In these cases, increases in acylated ghrelin levels, or increased GHSR1a expression, may enhance the sensitivity of the mesolimbic dopaminergic system where GHSR1a and D1R are coexpressed. Relapse may then be more a function of dopaminergic sensitivity, as mediated by the ghrelin receptor. If such hypotheses are correct, administration of a GHSR1a antagonist or a GOAT inhibitor may aid in drug addiction as well as in dieting. Pharmacological blockade of the GHSR1a or genetic ablation of this gene would result in decreased reward seeking behaviors. Indeed this seems to be the case, as *GHSR1a KO* mice display reduced food-anticipatory activity (Blum et al., 2009; Davis et al., 2011) and reduced food reward as measured by conditioned place preference (Zigman et al., 2005; Egecioglu et al., 2010; Perello et al., 2010; Chuang et al., 2011). Furthermore, locomotor responses to cocaine and alcohol self-administration are attenuated in GHSR1a- and ghrelin-deficient mice and rats (Jerlhag et al., 2010; Abizaid et al., 2011; Clifford et al., 2012). More research is needed, however, to specifically target the D1R-GHSR dimer and pinpoint its role in reward mechanisms, but it is clear that this would represent a fresh target for the treatment of addictions and the prevention of relapse.

### GHSR1a heterodimers as a target for the treatment of schizophrenia, obesity, and addiction

#### 5-HT_2C_ -GHSR1a dimer

In general, patients suffering from schizophrenia are treated with typical or atypical antipsychotic drugs. Typical antipsychotics generally block D2 dopamine receptor signaling, whereas atypical antipsychotics block both D2-like receptors and serotonin 5-HT_2A_ receptors. Both classes of antipsychotic medications have a number of side effects that include motor dysfunction (dyskinesia), obesity, and cardiovascular disease, as well as some endocrine abnormalities (for review, see Young et al., 2015). Interestingly, 5-HT_2C_ receptor modulators, and in particular 5-HT_2C_ agonists, have been identified as potential antipsychotic medications with fewer side effects (for review, see Rosenzweig-Lipson et al., 2012). Indeed 5-HT_2C_ agonists appear to regulate DA neurotransmission by decreasing DA cell activity in the VTA, a region also rich in GHSR1a expression (Di Giovanni et al., 2000). Additionally, treatment with 5-HT_2C_ antagonists enhances ghrelin-induced food intake, whereas treatment with lorcaserin, a 5-HT_2C_ agonist, decreases ghrelin-induced food intake (Schellekens et al., 2015). These behavioral effects may be mediated by internalization or changes in sensitivity of the 5-HT_2C_-GHSR1a receptor dimer.

Interestingly, pretreatment with the GHSR inverse agonist SP-analog enhances 5-HT_2C_ intracellular receptor signaling, supporting the notion that inverse agonists of the GHSR (and perhaps antagonists) may augment the effectiveness of 5-HT_2C_ in the treatment of schizophrenia. These effects, however, are likely sensitive to the timing of exposure of the dimer to the various ligands. Furthermore, given that the effects of 5-HT_2C_ agonists also include weight loss and a reduction in reward-seeking behaviors, the use of compounds that enhance 5-HT_2C_ signaling would also be clinically relevant for the treatment of obesity or to prevent relapse to drug abuse. This certainly warrants further exploration.

#### D2R-GHSR1a dimer

When Kern et al. (2012) administered the D2R agonist cabergoline following pretreatment with the GHSR1a neutral antagonist JMV2959, cabergoline’s anorexigenic effects were lost. Based on this result, the authors argue that antagonism of the ghrelin system may exacerbate rather than ameliorate obesity. Complicating the matter, administration of ghrelin still leads to cross-desensitization of dopamine-induced Ca^2+^ mobilization, and while ghrelin antagonism also reduces this mobilization, it is possible that concurrent cross-sensitization may also occur, which may possibly carry beyond the timeframe of bioavailability of such an antagonist. The balance between such effects, particularly over a prolonged timeframe, including effects at other GHSR1a monomers or GHSR1a heterodimers with other receptor types, must be taken into consideration.

#### MC3R-GHSR1a dimer

Being a system involved in satiety, the melanocortin system is a promising target for treatment of obesity and diabetes. While the melanocortin 4 receptor (MC4R) has often been the target for obesity treatment, to the best of our knowledge no dimerization between GHSR1a and MC4R has been identified. As was done in the study of D2R’s anorexigenic effects, the role of the GHSR1a-MC3R dimer may be teased apart by examining *GHSR KO* animals and MC3R agonists/antagonists. In addition to its role in feeding, evidence suggests that activation of MC3R may aid in insulin sensitivity and glucose control. While *MC4R KO* mice generally demonstrate a more severe diabetic phenotype, male *MC3R KO* mice nevertheless do display mild impairments in insulin sensitivity and glucose control, along with increased adiposity (Butler et al., 2000; Sutton et al., 2006). Administration of a melanocortin agonist to mice lacking MC4R but not MC3R improves hyperinsulinemia and homeostasis model assessment of insulin resistance (HOMA-IR) scores (Kumar et al., 2009). Furthermore, the coexpression of MC3R and GHSR1a in the hypothalamic arcuate nucleus suggests that this dimer may play a role in arcuate-mediated feeding behavior (Roselli-Rehfuss et al., 1993; Howard et al., 1996; Guan et al., 1997; Kistler et al., 1998; Jégou et al., 2000; Archer et al., 2004; Rediger et al., 2009).

Within the GHSR1a-MC3R dimer, the GHSR1a inverse agonist SP-analog eliminates amplification of MC3R signaling (Rediger et al., 2011), an effect likely due to the dependence on GHSR1a constitutive activity. While SP-analog has been shown to reduce weight gain, and hence likely aid in treatment of diabetes and obesity (Asakawa et al., 2003), when considering the dimer alone this attenuation may not be beneficial. GHSR1a neutral antagonists, however, do not eliminate basal activity, although the effects of such compounds on GHSR1a-MC3R have not been examined. Although there is no reason to believe that a neutral antagonist would directly affect MC3R signaling, the possibility remains that such an antagonist may lead to cross-sensitization or reduced internalization of the dimer, in effect amplifying MC3R signaling further through increased surface expression or sensitization.

#### CB1-GHSR1a dimer

While dimerization between the CB1 and GHSR1a receptors has not been fully confirmed, there is evidence suggesting that feeding responses to cannabinoids require intact GHSR1a receptors, and feeding responses to ghrelin require CB1 receptors. While one would suspect that coadministration of rimonabant, a selective CB1 antagonist, with a GHSR1a antagonist or inverse agonist may help to decrease appetite, or may reduce body weight with less side effects due to reduced dosage (Rinaldi-Carmona et al., 1994; Després et al., 2005), our current understanding of this potential dimer is too limited to allow us to speculate how such poly-drug treatment would affect signaling at the dimer. Alternatively, a combination of CB1 and GHSR1a agonists could be used to increase appetite and decrease nausea, for instance, in patients undergoing chemotherapy. Understanding the intracellular mechanisms underlying the interaction between these receptors may help improve CB1- or GHSR1a-based treatments to combat obesity.

## Conclusion

One of the big questions surrounding the ghrelinergic system has been the role of the ghrelin receptor in the brain. Demonstrating the presence of the ghrelin peptide centrally has remained difficult, and hence no definite ligand for GHSR1a has been identified in many areas of the brain. Following the identification of GHSR1a’s constitutive activity, investigators quickly started identifying dimerization partners of GHSR1a that occur under normal biological conditions with considerable evidence of activity resulting from the dimerized complexes. Thus, even in the absence of GHSR1a’s natural ligand, GHSR1a appears to have significant physiological effects via these protein−protein complexes.

Understanding how these dimers work, including how the signaling of each protomer is modified and how they affect different pathways and systems, is an area that should be enthusiastically studied by those interested in the development of novel pharmacological treatments. Being a receptor involved in many systems, including reward, feeding, and memory, GHSR1a represents an important target for the treatment of psychiatric and metabolic disorders. Due to its broad distribution, however, a wide range of side effects would be expected by targeting this receptor. While the technology does not yet exist, by narrowing the pharmacological target of drugs to specific dimers involving GHSR1a, it may be possible to significantly reduce side effects while targeting with more precision the culprits involved in the disorder being treated. Regardless of our ability to develop such drugs, by increasing our understanding of the changes induced by dimerization, one would hope that our ability to understand and treat disorders, such as through poly**-**drug treatments, will improve. Identification of the dimer partners presented here, which include 5-HT_2C_, MC3R, D1R, D2R, and CB1, represents only the beginning of our knowledge regarding dimerization involving GHSR1a. We can say with confidence that many more dimer partners will be identified within the near future.

## Synthesis

The decision was a result of the Reviewing Editor Karen Szumlinski and the peer reviewers coming together and discussing their recommendations until a consensus was reached. A fact-based synthesis statement explaining their decision and outlining what is needed to prepare a revision is listed below. The following reviewers agreed to reveal their identity: Tod Kippin and Nicholas Gilpin

### Synthesis of Reviews:

The review, entitled "Growth Hormone Secretagogue Receptor Dimers: A New Pharmacological Target" was deemed acceptable with moderate to major revisions to the text, following consultation with 2 reviewers with expertise in behavioral neuroendocrinology. Both reviewers felt that the manuscript was generally well-written and agreed that information provided should spark interest in the role for GSHRs in neuropsychiatry. One reviewer requested that the article contain more neuroanatomical information regarding the behavioral effects of GSHR1a stimulation, both alone and as dimers and suggested that a "big picture" figure to highlight the cooperative and competitive behavioral effects of monomer-dimer activation. This reviewer also suggested that some text be added to the review pertaining to how dimer effects of GSHR1a might impact the balance between the magnitude of the desired therapeutic effects and undesired off-target effects. The other reviewer provided a number of suggestions for improving the organization and flow of the text that includes minimizing the amount of text regarding information that has been reviewed elsewhere, or the text that is redundant or speculative. This reviewer also suggested the inclusion of a discussion of how ghrelin/GHSR agonists/antagonists might be employed for clinical treatment, as well as some discussion of the molecular interactions by GSHR and other receptors as they relate to specific clinical conditions. I concur with the assessments of this review by our expert reviewers and agree that major revisions are required in order to render it more acceptable for publication in eNeuro.

### Reviewer 1:

The present manuscript describes the role of the GHSR in intracellular signaling on its own and in a variety of molecular interactions with other receptors as well as the potential for stimulation of GHSRs as a therapeutic target for a wide-range of clinical conditions. This is an exciting and novel topic for which the molecular biology is particularly compelling as is the potential clinical applications for some of the discussed conditions. However, there are portions of the manuscript that simply repeat information reviewed by other sources and should be shortened (e.g. the requirements and detection of dimmers); contain high redundancy (for instance, the discussion of GHSR being an orphan receptor until its binding of ghrelin is mentioned multiple times as are the weakness of co-immunprecipitation strategies for dimer studies); or are just too speculative (see below for e.g.). As such the overall impact of the manuscript would benefit greatly by detailed editing and potential re-organization.

A general discussion of how ghrelin/GHSR agonists/antagonists would be employed for clinical treatment may be of use; this is touched upon at the end but only in a limited fashion. For instance, on lines 655-8, there is the suggestion of poly-drug treatment strategies for depression. Drug combinations can produce synergy which can be extraordinarily potent making this an intriguing direction. Would such a strategy be of wide use for multiple clinical conditions? If so what are the potential benefits and risks for such strategies? The discussion of oligomerization (page 3-8) is described elsewhere in detail (including cited references) and can be somewhat summarize here; for instance, the latter two sections are largely derivative of single sources.

Some sections appear to be highly speculative (e.g. "treatment of Parkinson's disease and Alzheimer's disease") and likely the section could be shortened to a single sentence indicating a potential use for ghrelin/GHSR agonists given the role of dopamine in these disorders and their management. Further, it seems odd to start off with these disorders because there is nothing cited to specifically implicate ghrelin therapeutics for these conditions.

Finally, the manuscript would be easier to navigate if the introduction was followed by discussion of the molecular interactions by GSHR and other receptor and then description of how these interactions may of relevance to specific clinical conditions. The current format has several clinical conditions visited multiple times (particularly obesity) and it might be worth considering how GSHR interactions with multiple other receptors may come into play in therapeutics.

### Minor concerns:

Line 39, sentence is overly-convulted; particularly inclusion of "among others"...

Line 365-6: Statement "In addition to this, similar situations that lead to drug relapse also lead to relapse in dieting individuals (Nair et al., 2011)" is confusing; does the latter relapse refer to non-drug relapse?

There are many abbreviations used in the text that are not found in the abbreviation list (given abbreviations are not always defined upon first use, it is critical that they be defined somewhere).

The title for Figure 4 should be reconsidered as the term "mutually opposite signal modulation" is unclear and/or does not reflect the description to follow. Similarly, for Figure 5, the figure legend should clarified if 5-HT2c-GHSR1a dimerization produces reduced Ca++ accumulation with (as appears to be implied by the figure) or without ligand for each receptor.

### Reviewer 2:

This manuscript aims to review the literature on growth hormone secretagogue receptor (GSHR1a) dimers as a promising and emerging pharmacotherapeutic target. GSHR1a forms dimers with D1R, D2R, Mc3R, 5-HT2cR, and potentially CB1R, and each of these dimers has different therapeutic implications based on the known distribution and behavioral effects of each of these receptors on their own. This is a very well written and organized review with good breadth for the likely readership of this journal, clear and useful figures, and suggestions for new potential avenues for future work in the field. My suggestions for improving this review are relatively minor as itemized below:

[1] The review includes an excellent explanation of GSHR1a dimerization with other receptors at the cellular and molecular level and also does a good job of explaining technical aspects of this type of work. However, I do believe that there is a gap in the review in tying molecular processes of GSHR1a dimerization to specific neuroanatomical regions (as alluded to on page 30) as a bridge to normal and pathological behaviors. The review would benefit from the addition of information on neuroanatomical regions as known and/or hypothesized loci for behavioral effects of the various dimer partners for GSHR1a, both alone and as dimers with GSHR1a. In the text, this information would likely be most useful added to each section as it currently stands. The addition of a figure would also be great to provide a "big picture" snapshot of where these various cooperative and competitive behavioral effects may be occurring.
[2]Related to the previous point, as mentioned by the authors, dimer effects of GSHR1a are "one small part of the puzzle" for many of these behaviors and disorders. The review would benefit from a bit more text on how this notion impacts the balance between desired therapeutic effects, "diluted" effects from dimer-mediated versus non-dimer effects, and undesired off-target effects (which are discussed some in terms of receptor specificity, but not in terms of anatomy, where the same dimer may have different effects in different brain regions).

## Author Response

Dear reviewers and editor,

We have read your comments and suggestions regarding our review paper “Growth Hormone Secretagogue Receptor Dimers: A New Pharmacological Target” and agree that the requested revisions improved the manuscript. We have addressed the suggestions to the best of our ability and summarize the changes below, along with the original comments from the reviewers.

### Reviewer #1

“The present manuscript describes the role of the GHSR in intracellular signaling on its own and in a variety of molecular interactions with other receptors as well as the potential for stimulation of GHSRs as a therapeutic target for a wide-range of clinical conditions. This is an exciting and novel topic for which the molecular biology is particularly compelling as is the potential clinical applications for some of the discussed conditions. However, there are portions of the manuscript that simply repeat information reviewed by other sources and should be shortened (e.g. the requirements and detection of dimmers); contain high redundancy (for instance, the discussion of GHSR being an orphan receptor until its binding of ghrelin is mentioned multiple times as are the weakness of co-immunprecipitation strategies for dimer studies); or are just too speculative (see below for e.g.). As such the overall impact of the manuscript would benefit greatly by detailed editing and potential re-organization.”

**Response:** As per your suggestion, we have replaced the section on requirements and detection of dimers with a reference to the original IUPHAR publication. We have also reduced redundancy mostly through reorganizing the paper (as discussed below in response to another comment), as well as through more minor editing such as removing repeated information (eg. Repetition of GHSR’s previous orphan status). We have also addressed the problem of the speculative nature of the paper, which is also discussed below.

“A general discussion of how ghrelin/GHSR agonists/antagonists would be employed for clinical treatment may be of use; this is touched upon at the end but only in a limited fashion. For instance, on lines 655-8, there is the suggestion of poly-drug treatment strategies for depression. Drug combinations can produce synergy which can be extraordinarily potent making this an intriguing direction. Would such a strategy be of wide use for multiple clinical conditions? If so what are the potential benefits and risks for such strategies? The discussion of oligomerization (page 3-8) is described elsewhere in detail (including cited references) and can be somewhat summarize here; for instance, the latter two sections are largely derivative of single sources.”

**Response:** We have added a section titled “General treatment approaches” appearing at the end of the article, before the concluding remarks. This section discusses how one can leverage the synergy observed in some dimers not only by amplifying an existing treatment, but also by reducing the dosage required, and hence possibly reducing potential side effects. This has also been addressed in our rewrite of some of the sections on dimer-based therapeutics, where we mention several poly-drug approaches. We have also shortened the sections on general oligomerization.

“Some sections appear to be highly speculative (e.g. "treatment of Parkinson's disease and Alzheimer's disease") and likely the section could be shortened to a single sentence indicating a potential use for ghrelin/GHSR agonists given the role of dopamine in these disorders and their management. Further, it seems odd to start off with these disorders because there is nothing cited to specifically implicate ghrelin therapeutics for these conditions.”

**Response:** In order to reduce speculation, we have removed the sections on D1R-GHSR1a’s possible role in Alzheimer’s, 5-HT2C-GHSR1a’s possible role in depression, anxiety, and stress, and D2R-GHSR1a’s possible role in antipsychotic-associated weight gain (although we have added an additional section pertaining to 5-HT2C-GHSR1a’s possible role in schizophrenia, along with considerable references to support our argument). In the section discussing D1R-GHSR1a and Parkinson’s, we have added additional references to support our argument. Overall, we have considerably shortened our discussion of suggested therapeutics, as these sections were the most speculative.

“Finally, the manuscript would be easier to navigate if the introduction was followed by discussion of the molecular interactions by GSHR and other receptor and then description of how these interactions may of relevance to specific clinical conditions. The current format has several clinical conditions visited multiple times (particularly obesity) and it might be worth considering how GSHR interactions with multiple other receptors may come into play in therapeutics.”

**Response:** We have reorganized the paper to discuss first the molecular mechanisms of all the dimers, followed by a separate discussion of possible ways to leverage these dimers to aid in therapeutics. This has helped reduce repetition and improve the flow of the paper. We believe that this suggestion and the modifications made have greatly helped in improving the quality of the paper.

“Line 39, sentence is overly-convulted; particularly inclusion of "among others"...”

**Response:** We have replaced this sentence with: “While these three components are all directly related to ghrelin, one cannot deny the complex interrelationships between ghrelin and the multitude of other systems beyond simply the typical feeding circuitry of this peptide.”

“Line 365-6: Statement "In addition to this, similar situations that lead to drug relapse also lead to relapse in dieting individuals (Nair et al., 2011)" is confusing; does the latter relapse refer to non-drug relapse?”

**Response:** We have replaced this sentence with: “Enhanced D1 receptor signaling would also explain why addicts are more prone to relapse when stressed or hungry, both conditions that augment ghrelin secretion and sensitivity.”

“There are many abbreviations used in the text that are not found in the abbreviation list (given abbreviations are not always defined upon first use, it is critical that they be defined somewhere).”

**Response:** All abbreviations have been added to the abbreviations list, and each abbreviation is now written out in full when first used.

“The title for Figure 4 should be reconsidered as the term "mutually opposite signal modulation" is unclear and/or does not reflect the description to follow.”

**Response:** We have replaced the term “mutually opposite signal modulation” with: “amplification of MC3R signaling and attenuation of GHSR1a signaling”

“For Figure 5, the figure legend should clarified if 5-HT2c-GHSR1a dimerization produces reduced Ca++ accumulation with (as appears to be implied by the figure) or without ligand for each receptor.”

**Response:** To make it clearer that the 5-HT2C ligand is not required, while the ghrelin ligand is, we have changed the description to read: “When dimerized with 5-HT2C, GHSR1a displays a 65% reduction in ghrelin-induced Ca2+ accumulation, with this effect not requiring the presence of a 5-HT2C ligand.”

### Reviewer #2:

“The review includes an excellent explanation of GSHR1a dimerization with other receptors at the cellular and molecular level and also does a good job of explaining technical aspects of this type of work. However, I do believe that there is a gap in the review in tying molecular processes of GSHR1a dimerization to specific neuroanatomical regions (as alluded to on page 30) as a bridge to normal and pathological behaviors. The review would benefit from the addition of information on neuroanatomical regions as known and/or hypothesized loci for behavioral effects of the various dimer partners for GSHR1a, both alone and as dimers with GSHR1a. In the text, this information would likely be most useful added to each section as it currently stands. The addition of a figure would also be great to provide a "big picture" snapshot of where these various cooperative and competitive behavioral effects may be occurring.”

**Response:** We appreciate your compliments regarding the paper. We agree that the addition of more neuroanatomical information would greatly benefit the paper. While we have added more information where possible, much of the current research regarding receptor dimerization have been conducted on cell lines. Where possible, we have added the source of these cell lines, and have gone through each section to add any additional neuroanatomical information that we may have missed. We have also added Figure 6, which summarizes all the GHSR dimers, the effects of these dimers as discussed in the paper, along with possible neuroanatomical locations important for these dimers. Each section discussing the molecular aspects of each dimer also has some information on the monomers.

“Related to the previous point, as mentioned by the authors, dimer effects of GSHR1a are "one small part of the puzzle" for many of these behaviors and disorders. The review would benefit from a bit more text on how this notion impacts the balance between desired therapeutic effects, "diluted" effects from dimer-mediated versus non-dimer effects, and undesired off-target effects (which are discussed some in terms of receptor specificity, but not in terms of anatomy, where the same dimer may have different effects in different brain regions).”

**Response:** We have added a new section title “General treatment approaches” before the concluding remarks. This provides a short discussion of taking advantage of the synergy in terms of therapeutics, particularly by allowing a reduction in dosage, including discussions regarding side-effects and various neuroanatomical regions. We have also tried to address this in our rewrite of the therapeutics section found in the second half of the paper.
